# Low-molecular-weight heparins utilization in pregnant and postpartum women: a real-world analysis in China (2016–2021)

**DOI:** 10.3389/fphar.2025.1519051

**Published:** 2025-03-24

**Authors:** Hao-Ran Liu, Kai Sun, Tao Zeng, Xian-Li Wang

**Affiliations:** Department of Pharmacy, Obstetrics and Gynecology Hospital of Fudan University, Shanghai, China

**Keywords:** enoxaparin sodium, nadroparin calcium, low-molecular-weight heparin, pregnancy, prescription data, venous thromboembolic disease

## Abstract

**Objectives:**

This study aimed to examine trends in low-molecular-weight heparin (LMWH) use for managing pregnancy-associated venous thromboembolism (VTE) and to explore the correlation between pregnancy-related VTE risk factors and LMWH prescription rates.

**Methods:**

This was a cross-sectional study that analyzed prescription data from pregnant and postpartum women using LMWH to manage VTE, collected during 2016–2021. Risk factors associated with VTE were analyzed. Separately comparing the number of prescriptions, prescription cost, defined daily doses (DDDs), and defined daily cost (DDC) of seven LMWH.

**Results:**

This study included 41,885 prescriptions, with the average age of patients being 32 ± 4.69 years old. The most common risk factors for VTE during pregnancy and the postpartum period in this study included advanced age (>35 years old), cesarean section, diabetes, miscarriage, and preterm birth, accounting for 28.61%, 25.60%, 18.34%, 17.31%, and 13.63% respectively. There was a 173% increase in LMWH prescription costs during the study period. In terms of number of prescriptions, prescription cost, and DDDs, enoxaparin sodium, nadroparin calcium, and low-molecular-weight heparin calcium consistently ranked in the top three from 2019 to 2021. In terms of DDC, low-molecular-weight heparin sodium, dalteparin sodium, and enoxaparin sodium were the lowest.

**Conclusion:**

From 2016 to 2021, both the number of prescriptions and the total prescription costs for the management of VTE during pregnancy and the postpartum period increased. Enoxaparin sodium, nadroparin calcium, and low-molecular-weight heparin calcium were the most common LMWH. Advanced age (>35 years old), cesarean section, diabetes, miscarriage, and preterm birth were the most common pregnancy-related VTE risk factors linked to LMWH prescription.

## 1 Introduction

Pregnancy and the postpartum period are well-established risk factors for venous thromboembolism (VTE). During pregnancy and the postpartum period, the incidence of VTE is approximately 0.06% (Morris et al., 2010), which is 4–5 times higher than that in non-pregnant women (American College of Obstetricians and Gynecologists’ Committee on Practice Bulletins—Obstetrics, 2018; Creanga et al., 2017). While the incidence of VTE during pregnancy is generally low, it is one of the leading causes of maternal mortality (American College of Obstetricians and Gynecologists’ Committee on Practice Bulletins—Obstetrics, 2018; Creanga et al., 2017). A study has shown that pulmonary embolism accounted for 9.20% of all maternal deaths in the United States in 2011–2013 (Creanga et al., 2017). Another study has shown that the prevention of VTE during pregnancy and postpartum period may reduce maternal mortality due to pulmonary embolism (Heit et al., 2005). The increased risk of VTE events caused by pregnancy-related physiological changes alone do not necessitate additional medical precautions (Sucker, 2020). In recent years, with the adjustment of China’s fertility policies and changes in marriage and childbirth concepts, the proportion of pregnant women aged 35 and above has increased. Among women in this age group, the prevalence of risk factors for venous thromboembolism (VTE), such as cesarean section, hypertension, heart disease, and obesity, is higher. The increase in these risk factors has directly led to a rise in the number of pregnant women with VTE risk factors (Rath et al., 2016). Consequently, the incidence of VTE has shown an upward trend over the past few decades (Kourlaba et al., 2016; Rath et al., 2016; Wang et al., 2025). This trend indicates that as the risk of VTE during pregnancy increases, the management of VTE are becoming increasingly important.

The Royal College of Obstetricians and Gynecologists (ROCG) in 2015, the American Society of Hematology in 2018, and the Chinese Society of Obstetrics and Gynecology in 2021 have all issued guidelines for reducing the risk of VTE during pregnancy and postpartum period. The guidelines suggested that VTE management should be stratified according to risk factor scores during pregnancy and the perinatal period. Low-molecular-weight heparins (LMWH) are recommended as the preferred anticoagulants for the prevention of VTE during pregnancy and postpartum period (Royal College of Obstetricians and Gynaecologists, 2015; Bates et al., 2018; Department of Obstetrics and Gynecology, Chinese Medical Association, 2021). LMWH represent a class of antithrombotic medications, which include dalteparin sodium, enoxaparin sodium, nadroparin calcium, parnaparin sodium, bemiparin sodium, and several others (Hao et al., 2019). Due to variations in manufacturing techniques, molecular weights, and *in vitro* anti-Xa activities among these LMWH, their pharmacokinetic, pharmacodynamic, and safety profiles *in vivo* exhibit variability. As a result, the clinical characteristics exhibited by a particular LMWH cannot be generalized to other LMWH (Frydman, 1996). Despite numerous studies on the management of VTE with LMWH, there is a significant gap in clinical practice applications in China that compare seven types of LMWH - including nadroparin calcium, enoxaparin sodium, dalteparin sodium, bemiparin sodium, parnaparin sodium, low-molecular-weight heparin calcium, and low-molecular-weight heparin sodium - in the management of VTE during pregnancy. This study aims to fill this research gap and also explores the frequency of risk factors that necessitate the use of LMWH for preventing VTE during pregnancy.

## 2 Data and methods

### 2.1 Data sources

All data were obtained from the database of the Prescriptions Analysis Cooperation Project, which is affiliated with the Hospital Pharmacy Professional Committee of the Chinese Pharmaceutical Association. This project involves multiple hospitals in nine cities, including Beijing, Shanghai, Guangzhou, Chengdu, Harbin, Hangzhou, Zhengzhou, Tianjin, and Shenyang. Outpatient prescriptions and inpatient medical orders for 10 working days in each quarter were randomly selected using the information system of each hospital, and compiled into the database. In this study, we extracted inpatient medical order information from the database for the years 2016–2021, covering 132 hospitals in nine cities (Supplementary Table S1). The extracted information included primary diagnoses, prescription codes, primary generic drug name, specifications, quantities, usages, dosages, medication costs, patient ages, genders, and cities. In this study, the term “primary diagnoses” refers to the specific diseases or conditions identified by the physician at the time of prescribing, as documented in the patient’s medical records. This study only analyzed the cost of the medications themselves, without examining the final payment status of these medication costs (e.g., expenses covered by medical insurance or out-of-pocket payments by individuals).

### 2.2 Inclusion and exclusion criteria

The extracted prescription data were cleaned. The inclusion criteria were as follows: 1) women with “pregnancy” or “premature delivery” or “abortion” or “postpartum” as the primary diagnosis; 2) prescriptions with “low-molecular-weight heparin” or “nadroparin” or “enoxaparin” or “dalteparin” or “parnaparin” or “bemiparin” as the primary generic drug name. The exclusion criteria were as follows: 1) age <18 years old; 2) age >49 years old; 3) prescriptions where LMWH is concurrently prescribed with other antithrombotic drugs, such as unfractionated heparin, aspirin, warfarin, or novel oral anticoagulants including rivaroxaban, apixaban, dabigatran, and edoxaban.

### 2.3 Study design

#### 2.3.1 Demographic analysis

We categorized all prescriptions into six age groups (18–24, 25–29, 30–34, 35–39, 40–44, 45–49 years) and nine city groups (Beijing, Shanghai, Guangzhou, Chengdu, Harbin, Hangzhou, Zhengzhou, Tianjin, and Shenyang), and compared the proportions of LMWH use for managing VTE during pregnancy across these age and city groups.

#### 2.3.2 Risk factor analysis

We identified risk factors based on those listed in relevant guidelines and included only those that matched specific disease names in our analysis. The risk factors considered included prenatal factors (e.g., diabetes, hypertension, twin pregnancy, eclampsia, uterine myoma, hypothyroidism, intrahepatic cholestasis, obesity, heart disease, assisted reproductive technology, renal disease, lupus erythematosus, thrombophilia, cancer, hypercoagulation and hyperviscosity, multiple pregnancies, antiphospholipid syndrome); postnatal factors (e.g., cesarean section, miscarriage, preterm birth, postpartum hemorrhage, stillbirth); and temporary factors (e.g., postoperation, infection, hyperemesis gravidarum, ovarian hyperstimulation syndrome, pelvic inflammation) (Royal College of Obstetricians and Gynaecologists, 2015; Department of Obstetrics and Gynecology, Chinese Medical Association, 2021; Shanghai Maternal and Child Safety Expert Committee et al., 2020; Linnemann et al., 2016; Queensland Health, 2020). We included only prescriptions with the aforementioned risk factors listed in the “primary diagnoses” field and compared the proportions of these risk factors (by comparing the risk factors against each other).

#### 2.3.3 Utilization analysis of different LMWH

We categorized all prescriptions into seven groups based on the type of LMWH used (Nadroparin Calcium, Enoxaparin Sodium, Dalteparin Sodium, Bemiparin Sodium, Parnaparin Sodium, Low-molecular-weight Heparin Calcium, and Low-molecular-weight Heparin Sodium). We compared the number of prescriptions, prescription cost, and defined daily doses (DDDs) along with their growth rates for different LMWH across the nine cities during the study period. DDDs = total annual dosage of drug/ DDD (Liu et al., 2022). The higher the DDDs, the higher the frequency of drug use. DDD refers to the assumed average maintenance dose per day for a drug used for its main indication in adults. The DDD for each drug was obtained from the ATC/DDD Index 2022 of the World Health Organization (ATC/DDD Index, 2022). For unlisted drugs, the appropriate dose for the indication was selected as the DDD based on the instructions for use and the usual clinical dosing (Liu et al., 2022).

#### 2.3.4 Economic analysis of different LMWH

We divided all prescriptions into seven groups (Nadroparin Calcium, Enoxaparin Sodium, Dalteparin Sodium, Bemiparin Sodium, Parnaparin Sodium, Low-molecular-weight Heparin Calcium, and Low-molecular-weight Heparin Sodium) and compared the defined daily cost (DDC) of each LMWH group, where DDC = annual cost to the patient/ DDDs of the drug (Liu et al., 2022). The higher the DDC, the higher the average daily cost of the drug.

### 2.4 Statistical methods

Data analysis was performed using Microsoft Excel 2021, SPSS software (version 25; SPSS Inc., Chicago, IL, United States), Python 3.9, and Pandas 1.4.0. These tools were used to clean, count, and classify the collected data, as well as to calculate DDDs and DDC. Continuous variables were expressed as mean ± standard deviation (SD), and comparisons between groups were performed using the Kruskal–Wallis test for non-normally distributed data. A p-value <0.05 was considered statistically significant.

### 2.5 Ethics statement

This study protocol was approved by the Ethics Committee of Obstetrics and Gynecology Hospital of Fudan University (approval number: 2023-01). Informed consent was waived due to the retrospective nature of the study.

## 3 Results

### 3.1 Demographic characteristic

A total of 44,304 prescriptions for LMWH were extracted from the database for hospitalized pregnant and postpartum women, and the data of 44,116 LMWH prescriptions remained after excluding ineligible data. Data with the same prescription number (n = 2,231) were combined into one prescription, and finally 41,885 prescriptions were included in this study. Based on the age stratification of the pregnant and postpartum women included in the study, the usage frequency of LMWH was highest among those aged 30–34 years, followed by those aged 25–29 years and 35–39 years (Table 1). It should be noted that the proportion of pregnant and postpartum women over the age of 35 years, who are considered to be of advanced maternal age, accounted for 28.61%. Of the prescriptions analyzed, 84.28% originated from Zhengzhou, Guangzhou, Shenyang, and Shanghai, while 15.72% were from Chengdu, Harbin, Beijing, Hangzhou, and Tianjin (Supplementary Table S2).

**TABLE 1:** Age distribution of pregnant and postpartum women using different low-molecular-weight heparins.

Age (years)	2016 (n = 2,993)	2017 (n = 3,712)	2018 (n = 7,121)	2019 (n = 8,763)	2020 (n = 10,250)	2021 (n = 9,046)	Total (n = 41,885)	Kruskal result statistic	P Value
mean ± SD	31.69 ± 4.66	32.13 ± 4.90	31.88 ± 4.99	31.86 ± 4.81	32.08 ± 4.55	32.15 ± 4.40	32.00 ± 4.69	70.01	<0.001
18–24 years (n, %)	123 (4.11%)	177 (4.77%)	382 (5.36%)	397 (4.53%)	418 (4.08%)	307 (3.39%)	1,804 (4.31%)		
25–29 years (n, %)	952 (31.81%)	1,013 (27.29%)	2099 (29.48%)	2,518 (28.73%)	2,462 (24.02%)	2,177 (24.07%)	11,221 (26.79%)		
30–34 years (n, %)	1,120 (37.42%)	1,384 (37.28%)	2,503 (35.15%)	4,414 (50.37%)	3,442 (33.58%)	4,016 (44.40%)	16,879 (40.30%)		
35–39 years (n, %)	628 (20.98%)	840 (22.63%)	1,573 (22.09%)	2,292 (26.16%)	1,818 (17.74%)	2064 (22.82%)	9,215 (22.00%)		
40–44 years (n, %)	160 (5.35%)	277 (7.46%)	519 (7.29%)	578 (6.60%)	540 (5.27%)	450 (4.97%)	2,524 (6.03%)		
45–49 years (n, %)	10 (0.33%)	21 (0.57%)	45 (0.63%)	51 (0.58%)	83 (0.81%)	32 (0.35%)	242 (0.58%)		

### 3.2 Risk factors associated with VTE

In this study, we included only prescriptions that had risk factors documented in the “primary diagnoses” field, and ultimately analyzed the VTE risk factors during pregnancy and postpartum period for 16,943 patients, which accounted for 40.45% of the prescriptions. Among these patients, only 1.21% required therapeutic use of LMWH due to VTE, while 98.79% received prophylactic LMWH based on VTE risk stratification. Among the patients receiving prophylactic LMWH, cesarean section, diabetes, miscarriage, preterm birth, hypertension, twin pregnancy, and eclampsia were the most common risk factors, with proportions of 25.60%, 18.34%, 17.31%, 13.63%, 9.90%, 7.77%, and 7.60%, respectively (Table 2).

**TABLE 2:** Risk factors associated with low-molecular-weight heparins use in pregnant and postpartum women from 2016 to 2021.

Risk factors		Number	Proportion
Prenatal	Diabetes	3,108	18.34%
	Hypertension	1,678	9.90%
	Twin pregnancy	1,316	7.77%
	Eclampsia	1,287	7.60%
	Uterine myoma	681	4.02%
	Hypothyroidism	399	2.35%
	Thrombosis	268	1.58%
	Intrahepatic cholestasis	248	1.46%
	Obesity	212	1.25%
	Heart disease	156	0.92%
	Assisted Reproductive Technology	145	0.86%
	Renal insufficiency/kidney disease	107	0.63%
	Lupus erythematosus	101	0.60%
	Thrombophilia	89	0.53%
	Cancer	88	0.52%
	Hypercoagulation and hyperviscosity	74	0.44%
	Multiple pregnancies	66	0.39%
	Antiphospholipid syndrome	32	0.19%
Postartum	Cesarean section	4,337	25.60%
	Miscarriage	2,932	17.31%
	Preterm birth	2,309	13.63%
	Postartum hemorrhage	770	4.54%
	Stillbirth	236	1.39%
Temporary	Postoperation	650	3.84%
	Infection	423	2.50%
	Hyperemesis gravidarum	144	0.85%
	Ovarian hyperstimulation syndrome	134	0.79%
	Pelvic inflammation	117	0.69%

### 3.3 Prescription cost, number of prescriptions, DDDs of LMWH

**FIGURE 1:**
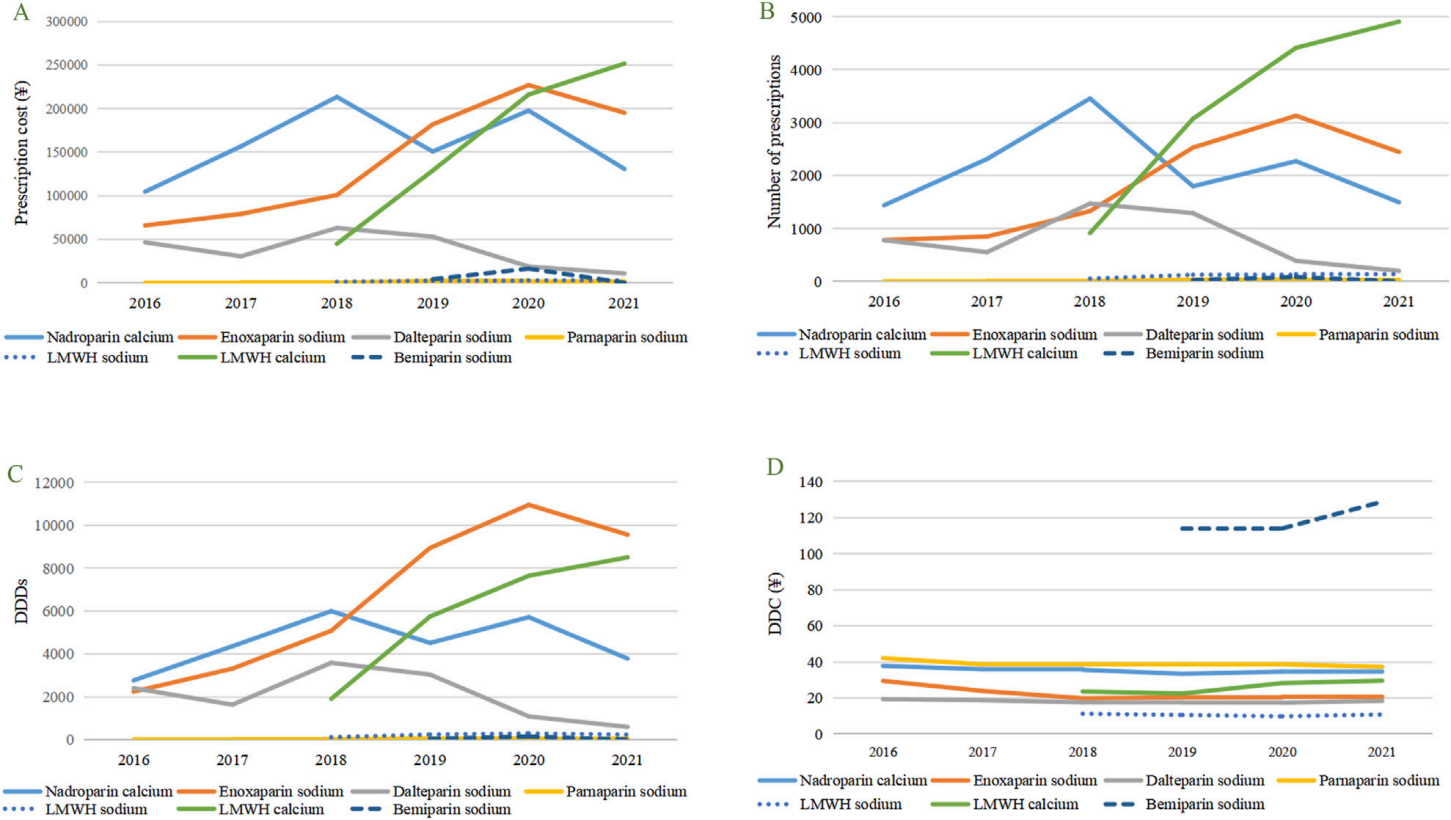
Utilization trends in terms of prescribing patterns of selected LMWH in pregnant patients from 2016 to 2021. **(A)** Prescription cost of different types of LMWH. **(B)** Number of prescriptions of different types of LMWH. **(C)** DDDs of different types of LMWH. **(D)** DDC of different types of LMWH. LMWH, low-molecular-weight heparin, DDDs, Defined daily doses, DDC, Defined daily cost.

During the study period, the total cost of prescriptions for LMWH increased annually, with a 172.80% increase from 2016 to 2021. From 2016 to 2018, nadroparin calcium, enoxaparin sodium, and dalteparin sodium consistently ranked among the top three in prescription cost, number of prescriptions and DDDs. However, from 2019 to 2021, this ranking underwent significant alterations, with low-molecular-weight heparin calcium surging to the top spot, joining enoxaparin sodium and nadroparin calcium to form the new top three in prescription cost, number of prescriptions and DDDs (Figures 1A–C). The prescription cost, number of prescriptions and DDDs for parnaparin sodium and bemiparin sodium were relatively low.

### 3.4 DDC of LMWH

From 2016 to 2021, there was little change in the DDC of the seven types of LMWH, and the ranking remained relatively stable, consistently ordered as follows: bemiparin sodium > parnaparin sodium > nadroparin calcium > low-molecular-weight heparin calcium > enoxaparin sodium > dalteparin sodium > low-molecular-weight heparin sodium (Figure 1D).

## 4 Discussion

This study collected prescription information for LMWH used during pregnancy and postpartum period from 132 hospitals in nine Chinese cities. We first analyzed the various risk factors, including age, associated with the use of LMWH in patients during pregnancy and postpartum period, and then compared the usage of different types of LMWH. By examining the prevalence of risk factors and prescribing trends of LMWH for managing VTE during pregnancy and postpartum period in China, our study aimed to refine clinical practices and enhance the cost-effectiveness of medication use, potentially informing future research and drug policy.

### 4.1 Management of pregnancy-related VTE: differences in age and region

In this study, the age distribution of pregnant and postpartum women using LMWH was as follows: 4.31% aged 18–24, 67.09% aged 25–34, and 28.03% aged 35–44, with pregnant and postpartum women aged 35 and above accounting for 28.61%. A study reported that the incidence of VTE in pregnant women during hospitalization and after discharge increased with age, and the risk for 15–24 years, 25–34 years, and 35–44 years was 363, 771, and 1,756 cases per 100,000 person-years, respectively (Sultan et al., 2013a). Another study also confirmed the highest risk of VTE in women 35 years and older (Sultan et al., 2013b). Therefore, the guidelines included ≥35 years of age as one of the risk factors for VTE assessment (Hao et al., 2019; Sultan et al., 2014). According to the latest fertility status of women of childbearing age released by the National Bureau of Statistics (National Bureau of Statistics of China, 2021), the fertility rates of women aged 25–29, 30–34, 35–39, and 40–44 years were 74.31‰, 45.31‰, 18.60‰, and 5.37‰, respectively. Although pregnant women aged 25–34 years have a lower risk of VTE compared to those aged 35–44 years, the larger population of women in the younger age group has led to a notably high proportion (67.09%) of them using low-molecular-weight heparin (LMWH) for VTE prevention in this study.

A study has shown that there are differences in the cognition and practice of preventing VTE during pregnancy across different regions (Ojukwu et al., 2023). In particular, economically developed regions pay more attention to the risk assessment and implementation of preventive measures for VTE during pregnancy (Ojukwu et al., 2023). This may explain why hospitals in Zhengzhou, Guangzhou, Shenyang, and Shanghai have higher average number of prescriptions of LMWH for preventing VTE during pregnancy and postpartum period, compared to those in Chengdu and Harbin. However, other factors require further investigation.

### 4.2 Analysis of specific risk factors related to VTE during pregnancy and postpartum period

The American College of Obstetricians and Gynecologists’ guidelines indicate that the management of thrombosis in pregnancy includes the treatment of acute thrombotic events and the prevention of an increased risk of thrombotic events. This study highlights that the majority of LMWH use during pregnancy and postpartum period (98.79%) was for VTE prevention, while only a small proportion (1.21%) was for therapeutic purposes due to VTE. This study conducted a categorical analysis of the risk factors associated with VTE during pregnancy and postpartum period, including prenatal, postpartum, and temporary factors. The results showed that among prenatal factors, diabetes, hypertension, twin pregnancy, and eclampsia had the highest frequencies, accounting for 18.34%, 9.90%, 7.77%, and 7.60%, respectively. These findings are consistent with previous studies, indicating that these factors play a significant role in the occurrence of pregnancy-related VTE (Royal College of Obstetricians and Gynaecologists, 2015; Department of Obstetrics and Gynecology, Chinese Medical Association, 2021; Shanghai Maternal and Child Safety Expert Committee et al., 2020; Linnemann et al., 2016; Queensland Health, 2020). For example, gestational diabetes, hypertension, and eclampsia may lead to vascular endothelial damage, thereby affecting coagulation function and increasing the incidence of VTE (Chen et al., 2019; Valero et al., 2023; Shi et al., 2019; Liu et al., 2024). Women with twin pregnancies have higher levels of blood D-dimer and a hypercoagulable state compared to those with singleton pregnancies, which increases the risk of VTE (Ren et al., 2020; Xu et al., 2023). Among postpartum factors, cesarean section, miscarriage, and preterm birth had the highest frequencies, accounting for 25.60%, 17.31%, and 13.63%, respectively. Post-cesarean section, factors such as wound pain and anesthetic effects may prevent mothers from getting out of bed shortly after delivery, leading to a prolonged hypercoagulable state (Royal College of Obstetricians and Gynaecologists, 2015). Additionally, cesarean section is associated with the activation of the coagulation cascade, further increasing VTE risk (Epiney et al., 2005). Recurrent miscarriage is partly attributed to a prethrombotic state caused by fetal-placental microcirculation disorders and a series of secondary pathological changes (Rodger, 2009). The increased risk of VTE in preterm birth may be related to prolonged bed rest and reduced activity (Zhu et al., 2021). Among the temporary factors, postoperative status and infections were relatively frequent, accounting for 3.84% and 2.50%, respectively. Postoperative status and infections may promote thrombosis through the release of inflammatory factors and activation of the coagulation system (Pastori et al., 2023). In previous studies, the incidence rates of pregnancy-related conditions were ranked as follows: diabetes > hypertension, preterm birth > eclampsia > hyperemesis gravidarum (Bailit, 2005; Feig et al., 2013; Tian et al., 2022). This ranking is largely consistent with the order of LMWH prescription rates observed in our study. Therefore, we speculate that there may be a certain correlation between LMWH prescription rates during pregnancy and the incidence of various VTE-related risk factors, although further research is needed to confirm this. Notably, our study revealed relatively low LMWH prescription rates for certain high-risk VTE factors (Queensland Health, 2020), including nephrotic syndrome (0.63%), heart failure (0.92%), thrombophilia (0.53%), ovarian hyperstimulation syndrome (0.79%), and hyperemesis gravidarum (0.85%). This may reflect either the lower incidence of these conditions during pregnancy or potential under-prescription of LMWH in these high-risk groups, indicating a possible gap between clinical practice and guideline recommendations. These findings underscore the importance of individualized VTE prevention for high-risk pregnant women, such as those with diabetes, hypertension, or multiple pregnancies. Future studies should further explore the interactions between risk factors and their combined impact on VTE occurrence, as well as investigate the reasons behind the under-prescription of LMWH in certain high-risk conditions.

### 4.3 Analysis of the use of different LMWH

This study found that enoxaparin sodium, nadroparin calcium, and low-molecular-weight heparin calcium were the most commonly used types of LMWH for VTE prevention during pregnancy and postpartum period, with low-molecular-weight heparin sodium, dalteparin sodium, and enoxaparin sodium being the types with lower average daily costs. As the preferred agents for the prevention and treatment of VTE during pregnancy and postpartum period, LMWH demonstrate significant advantages in terms of safety, efficacy, cost-effectiveness, and compliance. In terms of safety, LMWH significantly reduce the incidence of osteoporosis, thrombocytopenia, and allergic skin reactions compared to unfractionated heparin (UFH) (Greer, 2002), and exhibit excellent safety profiles for both mothers and fetuses (Deruelle et al., 2006). Among them, enoxaparin is associated with fewer bleeding events (Jacobson et al., 2020) and a lower incidence of allergic skin reactions (Greer and Nelson-Piercy, 2005), while nadroparin has a higher potential for causing allergic reactions compared to enoxaparin (Schindewolf et al., 2013). In terms of efficacy, a Chinese cohort study showed that enoxaparin sodium enabled more patients to achieve effective anti-Xa levels during pregnancy and the puerperium, with better treatment stability than nadroparin calcium and dalteparin sodium (Bai et al., 2021). Furthermore, cost-effectiveness analyses further support the advantages of LMWH, demonstrating their superior cost-effectiveness compared to UFH (Holzheimer, 2004) and indicating that the use of enoxaparin for VTE prevention in severe ovarian hyperstimulation syndrome is cost-effective, even during the first trimester of pregnancy (Wormer et al., 2018). In terms of compliance, enoxaparin sodium has shown good adherence during pregnancy (Patel et al., 2012). In summary, LMWH offer significant advantages in the prevention and treatment of VTE during pregnancy and postpartum period, with enoxaparin standing out in terms of safety, efficacy, cost-effectiveness, and compliance. The findings of this study are consistent with existing literature, further supporting enoxaparin as the preferred agent for the management of VTE during pregnancy and postpartum period.

## 5 Strengths and limitations

This study has several strengths: 1) Large Sample Size: Our study includes a large sample size from 132 hospitals across nine cities, which enhances the generalizability of our findings. 2) Real-World Evidence: By utilizing real-world data from the Prescriptions Analysis Cooperation Project, our study offers insights into actual clinical practices and prescription patterns. 3) Focus on High-Risk Population: We specifically analyze the use of LMWH in pregnant and postpartum women, a group known to be at higher risk for VTE. 4) Relevance to Clinical Practice: The findings are directly relevant to clinical decision-making and can inform guidelines for the management of pregnant and postpartum women at risk of VTE.

However, certain limitations should be acknowledged to provide a balanced interpretation of the results. First, the included data were collected from 132 hospitals in nine Chinese cities, and the information systems used by each hospital were not uniform, resulting in missing data. For example, approximately 59.55% of prescriptions were missing fields related to LMWH use in the original diagnosis, prompting us to exclude them from our analysis of VTE prevention risk during pregnancy. Unfortunately, due to limitations in the data extraction from our hospital information system, we were unable to fully capture the gestational age at which LMWH use was initiated or continued. Second, the data included in this study only represent the prescription patterns of LMWH during a single hospitalization of pregnant patients, and there is a lack of data on the use of LMWH by patients after the prescription, the outcomes of VTE prevention after using LMWH, and the pregnancy outcomes. This precludes analysis of the effects of different types of LMWH on pregnancy and VTE prevention outcomes associated with LMWH use in greater depth.

## 6 Conclusion

This study provides an in-depth analysis of the prescription patterns of LMWH for hospitalized pregnant and postpartum women in China from 2016 to 2021, offering valuable insights into clinical practices during this period. Our key finding is that prophylactic LMWH use significantly exceeded therapeutic use, which may reflect an increasing emphasis on the prevention of VTE during pregnancy. Additionally, our research has revealed a variety of risk factors associated with the prophylactic use of LMWH, including advanced age (>35 years old), cesarean section, diabetes, miscarriage, preterm birth, hypertension, twin pregnancy, and eclampsia, all of which are crucial for clinical doctors when assessing and managing the risk of VTE during pregnancy. Our findings suggest that enoxaparin sodium, nadroparin calcium, and low-molecular-weight heparin calcium were preferred LMWH for preventing VTE in pregnant women, based on their widespread use in clinical practice. These findings not only provide a basis for clinical decision-making but also offer direction for future research. Large-sample studies are needed to perform head-to-head comparisons of all LMWH types, explore the safety, efficacy, cost-effectiveness,and compliance of LMWH in the prevention of VTE in pregnancy. Through these efforts, we can anticipate improvements in the management of VTE during pregnancy, thereby enhancing maternal and child health outcomes.

## Data Availability

The raw data supporting the conclusions of this article will be made available by the authors, without undue reservation.

## Abbreviations

VTE, Venous thromboembolism; LMWH, Low-molecular-weight heparin; DDDs, defined daily doses; DDC, defined daily cost.
