# Supplementary material for: Low-molecular-weight heparins utilization in pregnant and postpartum women: a real-world analysis in China (2016–2021)
Source: Front Pharmacol. 2025 Mar 24;16:1519051. doi: 10.3389/fphar.2025.1519051 (PMC11973347; doi:10.3389/fphar.2025.1519051)
Supplement: Supplementary file 1 [file Table1.docx]

**SUPPLEMENTAL FILE**

**Title：****Low-molecular-weight heparins utilization in pregnant and postpartum women: a real-world analysis in China (2016–2021)**

Table S1. Cities and hospitals included in this study

| Region | Cities | First-Level Hospital | Second-Level Hospital | Third-Level Hospital | Total |
| --- | --- | --- | --- | --- | --- |
| First-tier cities | Beijing | 1 | 2 | 13 | 16 |
|  | Shanghai | 0 | 5 | 18 | 23 |
|  | Guangzhou | 0 | 0 | 15 | 15 |
| Non-first-tier cities | Harbin | 0 | 0 | 19 | 19 |
|  | Hangzhou | 0 | 0 | 12 | 12 |
|  | Chengdu | 0 | 2 | 9 | 11 |
|  | Shenyang | 0 | 0 | 12 | 12 |
|  | Tianjin | 1 | 1 | 7 | 9 |
|  | Zhengzhou | 0 | 2 | 13 | 15 |
| Total | | 2 | 12 | 118 | 132 |

Table S2. Geographical distribution of pregnant women using low-molecular-weight heparins from 2016 to 2021

| Region, n(%) | 2016 | 2017 | 2018 | 2019 | 2020 | 2021 | Total | Number of hospitals | Average number of prescriptions per hospital |
| --- | --- | --- | --- | --- | --- | --- | --- | --- | --- |
|  |  |  |  |  |  |  |  |  |  |
| Shanghai | 973  (32.51%) | 1,316  (35.45%) | 941  (13.21%) | 1,301  (14.85%) | 1,535  (14.98%) | 1,548  (17.11%) | 7,614  (18.18%) | 23 | 331 |
| Beijing | 87  (2.91%) | 91  (2.45%) | 137  (1.92%) | 224  (2.56%) | 445  (4.34%) | 405  (4.48%) | 1,389  (3.32%) | 16 | 87 |
| Guangzhou | 1,415  (47.28%) | 1,354  (36.48%) | 1,573  (22.09%) | 1,567  (17.88%) | 1,389  (13.55%) | 903  (9.98%) | 8,201  (19.58%) | 15 | 547 |
| Harbin | 6  (0.20%) | 14  (0.38%) | 38  (0.53%) | 179  (2.04%) | 256  (2.50%) | 140  (1.55%) | 633  (1.51%) | 19 | 33 |
| Tianjin | 12  (0.40%) | 145  (3.91%) | 352  (4.94%) | 450  (5.14%) | 419  (4.09%) | 384  (4.24%) | 1,762  (4.21%) | 9 | 196 |
| Chengdu | 18  (0.60%) | 14  (0.38%) | 38  (0.53%) | 147  (1.68%) | 201  (1.96%) | 189  (2.09%) | 607  (1.45%) | 11 | 55 |
| Hangzhou | 112  (3.74%) | 234  (6.30%) | 397  (5.58%) | 494  (5.64%) | 696  (6.79%) | 256  (2.83%) | 2,189  (5.23%) | 12 | 182 |
| Shenyang | 315  (10.52%) | 232  (6.25%) | 223  (3.13%) | 271  (3.09%) | 1,609  (15.70%) | 2,357  (26.06%) | 5,007  (11.95%) | 12 | 417 |
| Zhengzhou | 55  (1.84%) | 312  (8.41%) | 3,422  (48.06%) | 4,130  (47.13%) | 3,700  (36.10%) | 2,864  (31.66%) | 14,483  (34.58%) | 15 | 966 |
| First-tier citiesa | 2,475  (82.69%) | 2,761  (74.38%) | 2,651  (37.23%) | 3,092  (35.28%) | 3,369  (32.87%) | 2,856  (31.57%) | 17,204  (41.07%) | 54 | 319 |
| Non-first-tier citiesb | 518  (17.31%) | 951  (25.62%) | 4470  (62.77%) | 5671  (64.72%) | 6881  (67.13%) | 6190  (68.43%) | 24681  (58.93%) | 78 | 316 |

^a^First-tier cities: Beijing, Shanghai, and Guangzhou

^b^Non-first-tier cities: Zhengzhou, Shenyang, Tianjin, Hangzhou, Chengdu, Harbin
